# Supplementary material for: Identification of the SUT Gene Family in Pomegranate (Punica granatum L.) and Functional Analysis of PgL0145810.1
Source: Int J Mol Sci. 2020 Sep 10;21(18):6608. doi: 10.3390/ijms21186608 (PMC7554910; doi:10.3390/ijms21186608)
Supplement: Supplementary file 1 [file ijms-21-06608-s001.pdf]

Supplementary Materials

Identification of the *SUT* gene family in pomegranate (*Punica granatum* L.) and functional analysis of *PgL0145810.1*

Krishna Poudel#, Xiang Luo#, Lina Chen, Dan Jing, Xiaocong Xia, Liying Tang, Haoxian Li and Shangyin Cao *

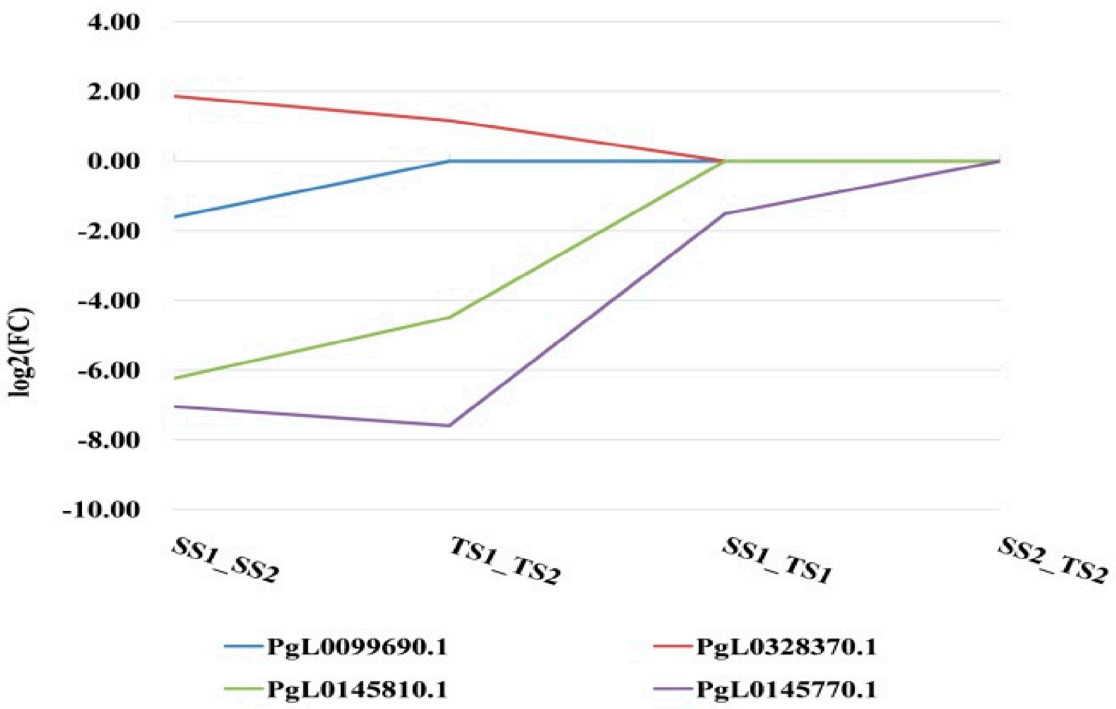

Figure S1. Expression of *SUT* genes during the development of seed in pomegranates
TS1: Seeds of 'Tunisia' at 60days after flowering (DAF); TS2: Seeds of 'Tunisia' at 120DAF; SS1:Seeds of 'Sanbai' at 60DAF; SS2: Seeds of 'Sanbai' at 120DAF; FC: fold change; SS1_SS2,TS1_TS2, SS1_TS1 and SS2_TS2: comparison group, text before '_' represents reference group,text after '_' represents treatment group.

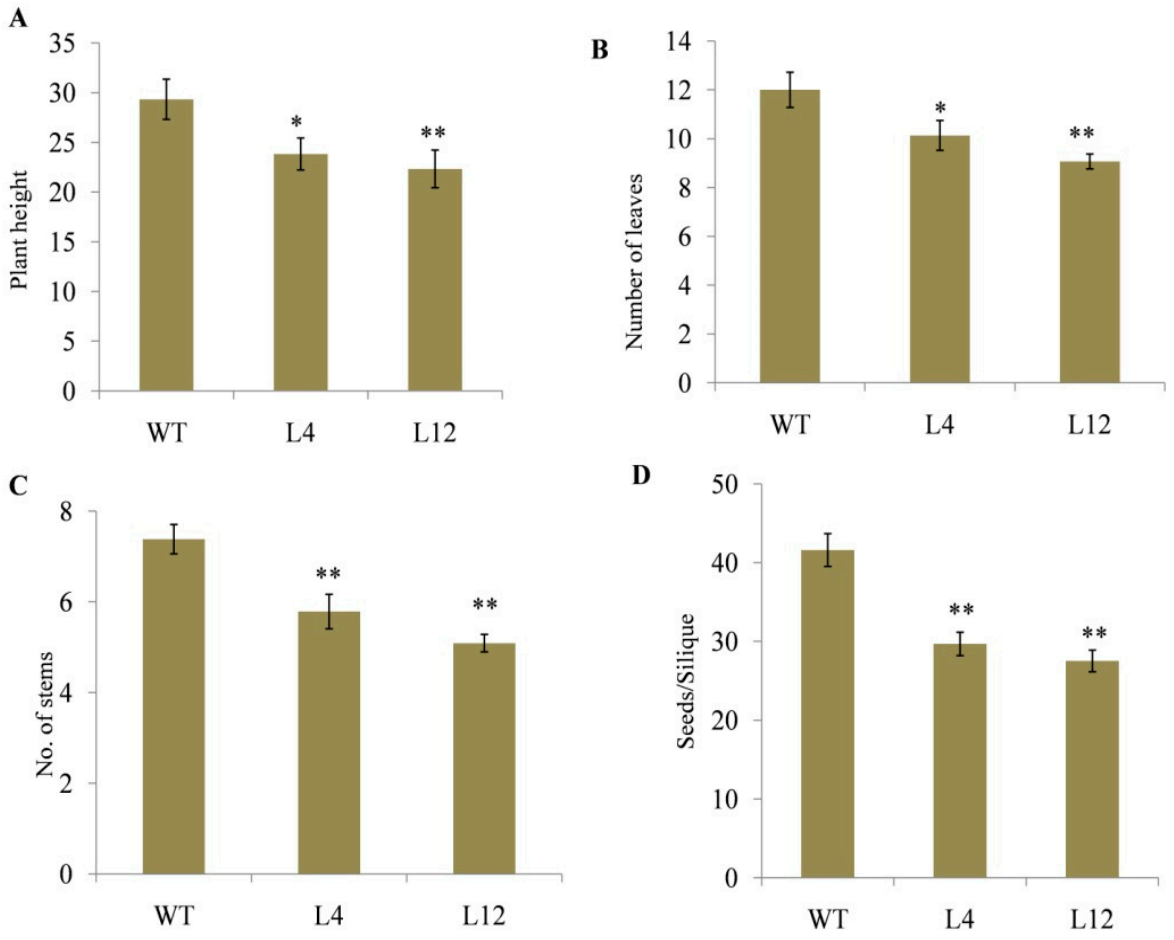

Figure S2. Growth parameters of wild-type (WT) *Arabidopsis* plants and transgenic *Arabidopsis* lines (L4 and L12). Data shown are plant height statistics of 42 days old seedlings (A), number of leaves of 35 days old seedlings (B), number of stems of 42 days old seedlings (C) and number of seeds per silique (D). Error bars indicates standard deviations from three repeats (n=3). Values are means ± SD (n = 3). * significant at $p < 0.05$ and ** significant at $p < 0.01$ probability levels.

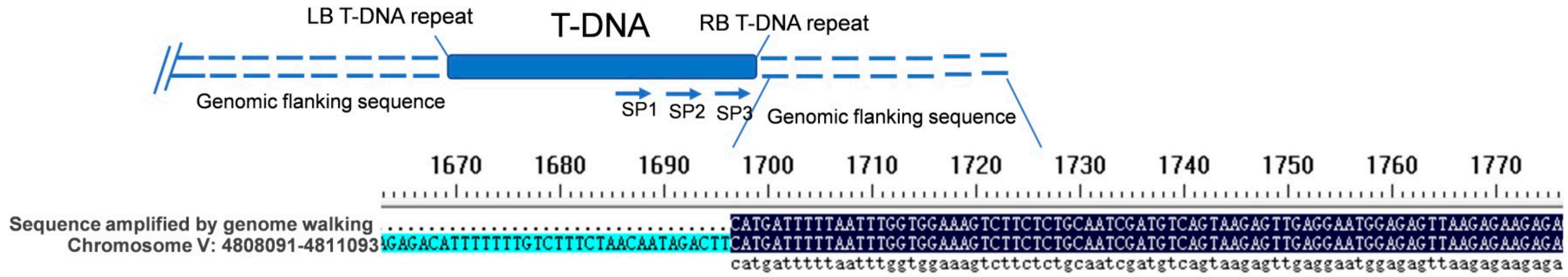

Figure S3. Schematic representation of the T-DNA insertion regions. Blue box: region of the T-DNA insertion; RB: right border; LB: left border; SP1, SP2 and SP3: specific primers; dotted lines: flanking plant DNA.

Table S1. The component of amino acid for *SUT* genes in pomegranate.

Protein_ name	*PgL0145770.1*	*PgL0099690.1*	*PgL0145810.1*	*PgL0181920.1*	*PgL0233780.1*	*PgL0237030.1*	*PgL0281820.1*	*PgL0328370.1*	*PgL0281810.1*	*PgL0281800.1*	Mean
Length	498	1236	581	515	1187	1251	92	507	155	289	631.1
M/W	53641.48	142986.14	60951.37	55655.52	133412.98	142472.24	10260.37	54848.93	18001.63	33693.48	70592.4
Ala	10.24	6.23	12.22	9.51	7.67	7.19	5.43	10.06	3.87	8.3	8.072
Cys	1.41	0.65	1.55	1.36	0.84	0.8	1.09	1.38	0	0.35	0.943
Asp	2.81	5.91	2.93	4.66	4.63	6	3.26	3.55	4.52	4.84	4.311
Glu	2.01	12.14	2.24	3.5	10.53	11.99	5.43	2.56	3.87	16.26	7.053
Phe	6.43	3.16	5.34	5.63	2.78	2.4	5.43	6.11	2.58	1.04	4.09
Gly	10.64	4.61	8.95	8.35	4.97	4.96	8.7	10.06	6.45	3.11	7.08
His	2.61	1.38	0.69	3.69	1.43	1.44	5.43	1.18	4.52	2.08	2.445
Ile	5.82	5.42	4.99	5.05	5.64	5.28	5.43	7.5	5.81	4.84	5.578
Lys	4.02	11.41	2.58	2.14	10.78	11.75	6.52	1.58	5.81	13.49	7.008
Leu	13.25	10.28	12.56	10.87	10.78	10.31	10.87	10.65	9.68	9.69	10.894
Met	3.01	1.78	2.07	2.52	2.11	2.72	2.17	2.76	3.23	2.08	2.445
Asn	3.01	4.13	3.79	5.05	4.8	3.68	5.43	2.96	3.87	3.46	4.018
Pro	5.42	2.18	7.23	5.05	1.94	2.4	3.26	5.92	5.81	1.38	4.059
Gln	3.01	5.34	2.07	4.08	6.32	4.4	2.17	2.96	2.58	4.84	3.777
Arg	3.21	6.72	3.96	4.08	4.38	5.52	6.52	5.72	7.74	7.96	5.581
Ser	5.62	6.31	9.12	9.51	7.33	6.95	7.61	6.71	9.68	6.57	7.541
Thr	3.61	4.37	5.85	3.3	3.71	3.84	3.26	5.33	6.45	2.42	4.214
Val	9.24	4.77	8.26	8.54	7.33	5.52	9.78	8.28	4.52	4.5	7.074
Trp	2.21	0.65	1.89	1.75	0.34	0.4	0	2.76	2.58	0	1.258
Try	2.41	2.59	1.72	1.36	1.68	2.48	2.17	1.97	6.45	2.77	2.56

Table S2. Raw quantitative real-time PCR (qRT-PCR) data for different pomegranate varieties.

PgL0145810.1		$2^{-\Delta\Delta CT}$			**Avg.**
**Varieties**	**Type of variety**	**1**	**2**	**3**	
**ZSL8**	Soft seeded	1.8921	1.6245	1.9318	1.8161
**Jiu Zi Hong**	Hard seeded	0.6029	0.5069	0.4383	0.5160
**Yi3**	Soft seeded	0.9054	0.9180	0.9771	0.9335
**Yi 2000-1**	Hard seeded	0.0834	0.0919	0.0645	0.0799
**Sanbai**	Hard seeded	0.0311	0.0397	0.0309	0.0339
**Tunisia**	Soft seeded	0.5573	0.5236	0.6402	0.5737

Table S3. Raw quantitative real-time PCR (qRT-PCR) data for different transgenic *Arabidopsis* lines.

PgL0145810.1	$2^{-\Delta\Delta CT}$			**Avg.**
**Transgenic lines**	**1**	**2**	**3**	
**2**	5.5148	1.0594	2.7574	3.1106
**3**	5.5918	3.6637	2.2868	3.8475
**4**	0.4362	0.2558	0.2918	0.3280
**5**	0.4233	0.4897	0.3035	0.4055
**6**	4.8680	4.9703	2.7194	4.1859
**7**	7.0779	6.6039	5.6699	6.4506
**9**	3.7407	3.8193	3.3948	3.6516
**10**	2.4116	1.8660	1.5691	1.9489
**11**	0.4919	0.3780	0.3430	0.4044
**12**	9.7360	8.4757	8.5346	8.9155
**13**	8.6139	6.5735	4.1315	6.4396

*** Note; (2,3,4,5,6,7,9,10,11,12,13): Transgenic *Arabidopsis* Lines**

Table S4. Raw quantitative real-time PCR (qRT-PCR) data for different tissues of both transgenic *Arabidopsis* lines.

PgL0145810.1		$2^{-\Delta\Delta CT}$			**Avg.**
**Tissues**		**1**	**2**	**3**	
**Leaf**	**L4**	1.3348	0.8991	0.8331	1.0223
	**L12**	1.0942	1.4539	2.0279	1.5253
**Stem**	**L4**	2.9759	1.5404	3.9540	2.8234
	**L12**	2.9553	4.6697	2.3839	3.3363
**Flower**	**L4**	1.2226	1.9318	2.5140	1.8895
	**L12**	2.4509	1.0594	0.9159	1.4754
**Silique**	**L4**	1.8108	1.2454	1.5122	1.5228
	**L12**	1.3534	1.3073	2.1987	1.6198

*** Note; L4: Transgenic Arabidopsis Line L4**
L12: Transgenic Arabidopsis Line L12

Table S5. The flanking sequence amplified by genome walking.

**Flanking Sequence**
ttttttgcccataatgactacactttaaacgtcttctttatctatagagaaatcaggctcatctggtttg<br>aatagttgaggcatcttcaaactggtggaagatccatctttcttaccagtactactacttgaagcctttt<br>gatttgttcctcttctcgtgttcgccgcaaatttagatgctaagatcgttgctcccagatttgtgttgtc<br>cattgatccgccttcctcgtcacttccatagtaatactctctagtttcttcgtcttcttcattgtaacct<br>gtttcatccgggtaatagtatccgctactctcgtgaaggctgagctctttcgcaagctttcttcgcttgt<br>atcttctccatgcagattggacaaaacatgctccccatgctctccattgatgtgagtagtacctgaaagc<br>gtgttgaagctttttgctttgaagacgtttgaactgatgagcaacaaacttaagatcctctgcgcttaat<br>gcaaaggcttctacttcagagagggcacggacgcttcgtgttgaagacggaagattgagtgtagagtttg<br>gcattaaggcccatgtaagaagttcttccccacagaaatcgccaggtcgtagagtagtggagttgaagaa<br>accagatcttcctccatttgttgttgagctctctatttgtcctcggatcacaaatagcatctcattcacc<br>ggatcaccttcacggaatatgtatgtcccggccgtgcttaaagatgagacaaggcatccgcatatagcat<br>caagaagttgatcatccatttgagagaagaacgggacctgcacttaccacaacaatgtgacttctgaatt<br>gaaaaacaatcaaatccataacagtttttgatgactgagttgatgtatgtacccggcgaacaagggagag<br>acaaagatgacgttggatttcacggcgtaaatctgtaggtaatgaatggaggattgattcttcatcgaca<br>cctctggttgcaagccatttgtattggacaaatcttcggacacgttcttgaagctctggaggtagttgac<br>gatgtctcatccattcttcagtgtctcttctcttaactctccattcctcaactcttactgacatcgattg<br>cagagaagactttccaccaaattaaaaatcatgaagtctattgttagaaagacaaaaaaatgtctctgta<br>ttgaaaagttttgcagagtgatgcataagctctgaatcttacttgcatgttaccaatcaggagtgtgaag<br>agaatcaacccaaaaatacaaattgttatacagaacaaggtctcgccaaggtatacacttgtggttatat<br>tctgtccataagaactgtgaaacaaaacctttattaactaaaaacttcgata

Table S6. Primers for cloning and for quantitative real-time PCR (qRT-PCR) assay of *PgL0145810.1*.

Primer name	Sequence (5'-3')	Function
PgL0145810.1-F	CTTTCTAGAATGGCGAAAGTTGAGGAGG	Gene cloning
PgL0145810.1-R	CTTGAGCTCTCAGTGCTGAGACTTCCAT	Gene cloning
Q *PgL0145810.1*-F	ATCACCAAGCTGGCTGACTC	qRT-PCR
Q *PgL0145810.1*-R	GCACCGGAGGAATTGGAGAA	qRT-PCR
Q *Pg*Actin-F	AGTCCTCTTCCAGCCATCTC	qRT-PCR
Q *Pg*Actin-R	CACTGAGCACAATGTTTCCA	qRT-PCR
Q Actin-F	GGTAACATTGTGCTCAGTGGTGG	qRT-PCR
Q Actin-R	AACGACCTTAATCTTCATGCTGC	qRT-PCR
RB-SP1	AGACTCTAATTGGATACCGAGG	Thermal asymmetric interlaced PCR
RB-SP2	TTAAACTCCAGAAACCCGCGGCTG	Thermal asymmetric interlaced PCR
RB-SP3	CTCCGCTCATGATCAGATTGTCGTT	Thermal asymmetric interlaced PCR
